# Herbal formula, Scutellariae radix and Rhei rhizoma attenuate dimethylnitrosamine-induced liver fibrosis in a rat model

**DOI:** 10.1038/srep11734

**Published:** 2015-07-02

**Authors:** Tai-Long Pan, Pei-Wen Wang, Chun-Hsun Huang, Yann-Lii Leu, Tung-Ho Wu, Yun-Ru Wu, Jyh-Sheng You

**Affiliations:** 1 School of Traditional Chinese Medicine, Chang Gung University, Taoyuan, Taiwan; 2 Chinese Herbal Medicine Research Team, Healthy Aging Research Center, Chang Gung University, Taoyuan, Taiwan; 3 Research Center for Industry of Human Ecology, Chang Gung University of Science and Technology, Taoyuan, Taiwan; 4 Graduate Institute of Natural Products, Chang Gung University, Taoyuan, Taiwan; 5 Division of Cardiovascular Surgery, Veterans General Hospital, Kaohsiung, Taiwan; 6 Graduate Institute of Traditional Chinese Medicine, Chang Gung University, Taoyuan, Taiwan; 7 Department of Chinese Internal Medicine, Chang Gung Memorial Hospital, Taoyuan, Taiwan

## Abstract

The bioactive components extracted from Scutellariae radix and Rhei rhizoma (SR) have been commonly used to treat liver diseases. The aim of this study was to verify the underlying mechanisms and antifibrotic effects of ethanol extract from the herbal combinatorial formula (SRE) in a dimethylnitrosamine (DMN)-administered rat model, with functional proteome tools. Our results indicated that the hepatic collagen content and alpha-smooth muscle actin expression were obviously alleviated by treatment with SRE. Comprehensive proteomics revealed global protein changes, and the network analysis implied that SRE application would attenuate oxidative stress and cytoskeleton dysregulation caused by DMN exposure. Next, marked downregulation of antioxidant enzymes mediated by DMN treatment was restored in the presence of SRE, while SRE treatment contributed to decreased MDA content. Moreover, protein carbonylation and DNA adduction induced by oxidative stress finally leading to liver injury were also reduced under SRE administration. These findings demonstrate that SRE could effectively prevent hepatic fibrosis mainly through regulating the redox status, and subsequently modulating the modification of intracellular molecules. Our experiments might help in developing novel therapeutic strategies against oxidation-caused liver diseases.

Hepatic fibrosis is caused by a wound-healing response to chronic liver injury resulting from viral hepatitis and metabolic and alcoholic liver diseases^1^. During fibrogenesis, persistent production of growth factors, cytokines, proteolytic enzymes and angiogenic factors stimulate the extracellular matrix (ECM) deposition, a proves which destroys normal tissue’s architecture^2^. Untreated fibrosis may progress to irreversible cirrhosis and induce molecular events that ultimately lead to organ failure and death^3^. Thus, developing antifibrotic treatments is urgently needed.

Various methods, including bile duct ligation, ethanol feeding and the use of hepatotoxin reagents such as dimethylnitrosamine (DMN) could be applied to induce experimental animal models of hepatic fibrosis^4^,^5^; however, this procedure has been reported to be harmful to the liver due to increased oxidative stress^6^,^7^. Previous reports also indicate that DMN rapidly induces liver fibrosis within two weeks, resulting in the production of reactive oxygen species (ROS), followed by the oxidation of lipids, proteins and DNA^8^. In addition, the DMN-induced hepatic fibrosis model presents most of the clinical manifestations observed in human liver fibrosis, including ascites, overproduction of ECM and histopathological changes^9^. This model is also stable after termination of DMN administration; it is also a reliable tool for screening antifibrotic agents^10^,^11^. We therefore chose an experimental DMN-induced liver fibrosis model for the current study.

Several traditional Chinese formulae including “Da-Chaihu-Tang” and “San-Huang- Xhi-Xin-Tang” have been used for liver disease therapy^12^,^13^. Moreover, liver fibrosis is manifested as inflammation and blood stasis based on TCM theory. We propose it is reasonable to use the combination of two important ingredients, Scutellariae radix (*Scutellaria baicalensis* Georgi; Huangqin in Chinese) and Rhei rhizoma (*Rheum palmatum* Linne, Da Huang in Chinese), which are considered for heat-clearing, qi regulation and detoxifying to attenuate liver fibrosis and reduce cytotoxicity with respect to unilateral medicine. Scutellariae radix is a widely used herb in traditional Chinese medicine with anticancer, antiviral and anti-inflammatory properties^14^. Recent investigations have shown that Scutellariae radix can inhibit cancer cell growth and attenuate inflammation^15^. Some studies also demonstrate that Scutellariae radix can effectively suppress fibrosis and lipid peroxidation^16^,^17^. Rhei rhizoma, commonly known as Chinese rhubarb, is a traditional Chinese medicine used for treating liver diseases, constipation and inflammation^18^,^19^. Rhei rhizoma and its anthraquinone constituents can also exert a protective effect against carbon tetrachloride (CCl_4_)-mediated liver injury^20^. With this in mind, we used the formula made of Scutellariae radix and Rhei rhizoma to reduce hepatotoxicity caused by DMN.

A great number of proteins and molecules will be changed in regard to quantity and quality in the development of liver fibrosis^21^. Thus, high throughput proteome tools combined with bioinformatics for data mining promise a superior pathway for large-scale screening and differentially identifying protein profiles that reflect various stages of disease or therapeutic effects^20^. Moreover, a functional “signature network” using MetaCore™ pathway analysis tools generates global cellular mechanisms underlying differences in protein levels and predict signaling pathways based on the architecture, in order to represent biological functionality and integration of molecular and clinical information^22^,^23^.

In this study, we aimed to discover the protein targets and molecular mechanisms by which SRE could effectively ameliorate liver fibrosis. Our results may accelerate the development of new therapies for patients with liver fibrosis.

## Results

### Characteristics of pure compounds from herbal formula SRE

**Figure 1:**
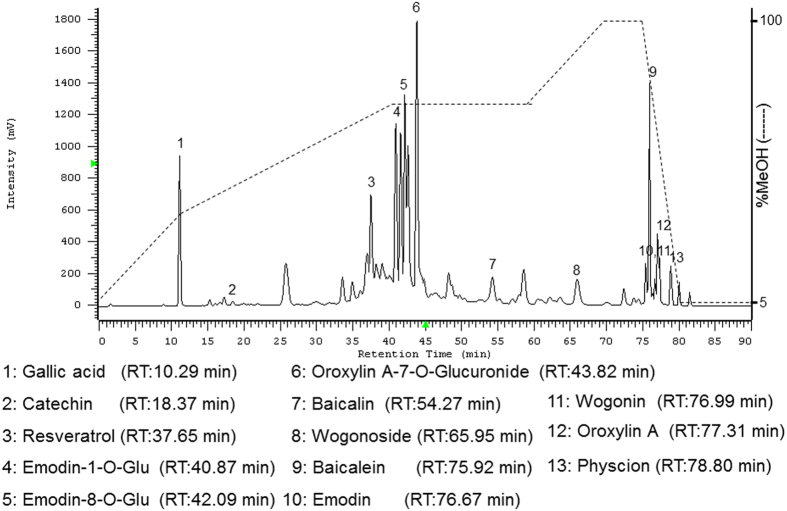
HPLC-UV 280nm chromatograms of ethanol extract produced from SRE. The quantification of samples was performed using a Hitachi L-2130 HPLC system comprising a gradient pump and the column used the Mightysil RP-18, 5 μm (250 × 4.6 mm) maintained at ambient room temperatures.

Thirteen constituents were identified by comparing the retention time with reference standard. The main compounds of SRE were as follows: gallic acid, catechin, resveratrol, emodin-1-O-Glu, emodin-8-O-Glu, oroxylin A-7-O-Glucuronide, baicalin, wogonoside, baicalein, emodin, wogonin, oroxylin A and physcion (Fig. 1).

### Effects of herbal combinatorial formula (SRE) upon liver function, pathological changes and α-SMA level of DMN-induced liver fibrosis in rats

**Figure 2:**
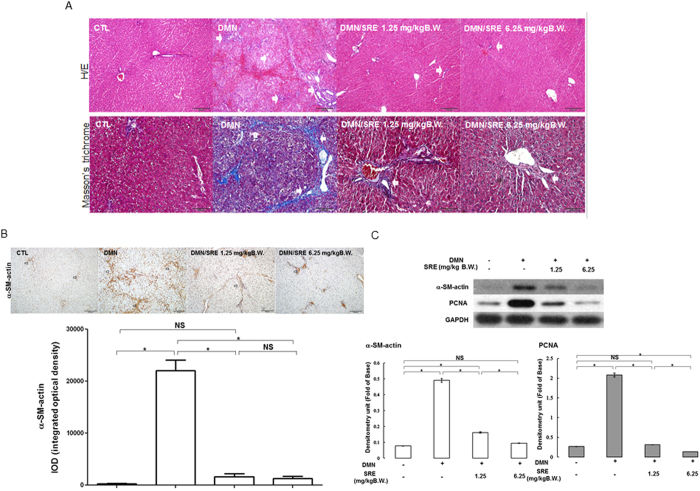
Histological analysis and assessment of cellular damage in rat liver tissues (**A**) Hematoxylin and eosin (H&E) staining and Masson’s trichrome staining. Original magnification: 100× and 200×. (**B**) IHC analysis revealed α-SMA expression in control (CTL), DMN, DMN/SRE 1.25 mg/kgB.W. and 6.25 mg/kgB.W. samples; the regions with differently expressed α-SMA are indicated by arrows. The intensity was calculated using the Image Pro-Plus 4.5 computer program. IOD: integrated optical density. (**C**) α-SMA was detected in whole liver tissue lysates by Western blot analysis, and the quantified results were indicated by the bar chart. GAPDH was used as an internal control. These results are representative of the rats used in each group (n = 3).

Here, histological changes in the liver tissue of rats were examined to evaluate the protective effect of SRE against hepatic fibrosis *in vivo*. Intact lobular architecture with central veins and radiating hepatic cords was observed in the normal control, while DMN exposure led to severe hepatic damage, demonstrated as increased collagen synthesis, sinusoidal congestion, massive necrosis of hepatocytes and inflammatory infiltration (Fig. 2A, indicated by arrows). Conversely, 1.25 and 6.25 mg/kg B.W. of SRE significantly attenuated the steatosis, fibrosis and collage expression.

α-SMA (α-smooth muscle actin) is a typical marker of liver fibrogenesis. Immunohistochemical staining revealed that the expression of α-SMA expression was prominent in the model liver, whereas a slightly positive signal of α-SMA was observed in the liver of the control rat. Administration of SRE at either low or high dose evidently diminished the expression of α-SMA in the liver (Fig. 2B). As expected, Western blotting analysis also confirmed that DMN exposure would stimulate α-SMA expression, in comparison to the control, whereas a much lower level of α-SMA was observed in the SRE-treated group with respect to DMN-treated, indicating that SRE treatment could effectively eliminate hepatic fibrosis induced by DMN. Again, similar trends in hepatic collagen content were observed by determining the HyP level (Table 1). Moreover, DMN-promoted protein expression of proliferating cell nuclear antigen (PCNA) indicating proliferation of hepatic stellate cell (HSC) caused by hepatocyte necrosis was significantly attenuated in the SRE-treated groups in a dose-dependent manner (Fig. 2C). These results indicate that SRE administration could effectively eliminate hepatic fibrosis and ameliorate abnormal morphologic changes induced by DMN in the animal model.

To assess the effect of SRE on liver function, serum aminotransferases and total bilirubin were determined. As depicted in Table 1, DMN resulted in a significant increase in plasma alanine transaminase (ALT), aspartate transaminase (AST) and total bilirubin levels with respect to the control samples, whereas SRE treatment effectively reduced these parameters. On the other hand, DMN led to downregulation of albumin, while the level of albumin was restored under SRE application in a dose-dependent manner. Moreover, 1.25 and 6.25 mg/kg B.W. of SRE show no significant impact on the normal functioning of kidney and liver as the levels of ALT, AST and total bilirubin were within the normal range, indicating that SRE at working concentrations lacks toxicity.

### Proteomic analysis and identification of differential protein targets

**Figure 3:**
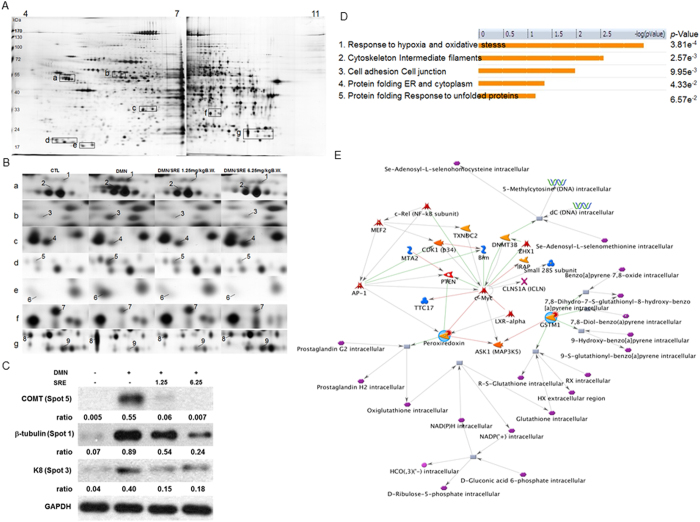
(**A**) Typical 2-DE protein profiles of rat liver samples. (**B**) Close-up figures show changes in the levels of protein expression among various groups. Each spot volume was determined, and their intensities were quantified by sliver-stained 2-DE (Nonlinear Progenesis software).The protein spots found to be significantly different in volume are indicated by Arabic numbers. (**C**) The protein levels of COMT, β-tubulin and K8 were assessed by Western blot analysis. GAPDH was used as an internal control and the relative expression to GAPDH is shown at the bottom. (**D**) Top-ranked pathways from the GeneGO MetaCore^™^ pathway analysis. Pathways were ranked according to p values, and bars represent the inverse log of the *p* value. (**E**) Biological network analyses of differentially expressed proteins using MetaCore™ mapping tools. Nodes represent proteins and lines between the nodes indicate direct protein–protein interactions. The various proteins on this map are indicated by different symbols representing the functional class of the proteins.

To uncover particular proteins associated with the protective effects of SRE against DMN-caused liver fibrosis and potent therapeutic targets, we used two-dimensional polyacrylamide gel electrophoresis (2-D PAGE) analysis to comprehensively characterize protein changes arising from rats that underwent different treatments. Fig. 3A demonstrates a representative set of silver-stained gels from reproducible gel patterns of three independent experiments. More than 1,500 protein spots are shown in each gel. Nine proteins with significant changes in protein volume were further identified by a peptide mass fingerprint (PMF), as displayed by the expanded plots in Fig. 3B, and indicated by Arabic numerals. Of these, cytoskeleton proteins and enzymes responsible for oxidative stress were identified. Table 2 summarizes the results of mass spectrometric analyses and suggested functions of identified proteins. Of these proteins, glutathione S-transferase Mu 2 (spot 8) and glutathione S-transferase Mu1 (spot 9) were unambiguously characterized by MS/MS (LIFT mode) analysis of the mass peptide fragments as indicated in supplementary Fig. 1S. To verify the results revealed by the proteomics, Western blotting was used to determine the expression of the associated proteins. We detected remarkable increases in the levels of catechol O-methyltransferase (COMT), β-tubulin and K8 for the DMN-exposed sample compared to the control, whereas these proteins were noticeably downregulated in a dose-dependent fashion under SRE treatment. A similar trend of the induced protein expression levels was shown in the results as they appeared in 2-D analysis (Fig. 3C).

### Functional network analysis

To further elucidate the relationship of differentially expressed proteins revealed by the proteomics analysis to their significance in the possible mechanisms related to the antifibrotic effects of SRE, the targeted proteins were further analyzed with the MetaCore™ analytical tool. The biological networks were built based on uploaded proteins, and the biological process was appointed to each network, as shown in Fig. 3D. Protein–protein interaction networks were involved in the following statistically significant networks: response to hypoxia and oxidative stress (*p* = 3.81 × 10^−4^), cytoskeleton intermediate filaments (*p *= 2.57 × 10^−3^), cell adhesion and cell junction (*p* = 9.95 × 10^−3^), protein folding ER and cytoplasm (*p *= 4.33 × 10^−2^), protein folding response to unfolded proteins (*p *= 6.57 × 10^−2^). The *p*-value indicates the significance of the assigned GO process on the basis of assembly size, as compared with the subnetworks derived from the input protein list. As shown in Fig. 3E, the enriched process network indicates that proteins differentially expressed after various treatments were primarily associated with oxidative stress.

### The Effect of SRE on antioxidant enzymes and oxidative modification of biological molecules

**Figure 4:**
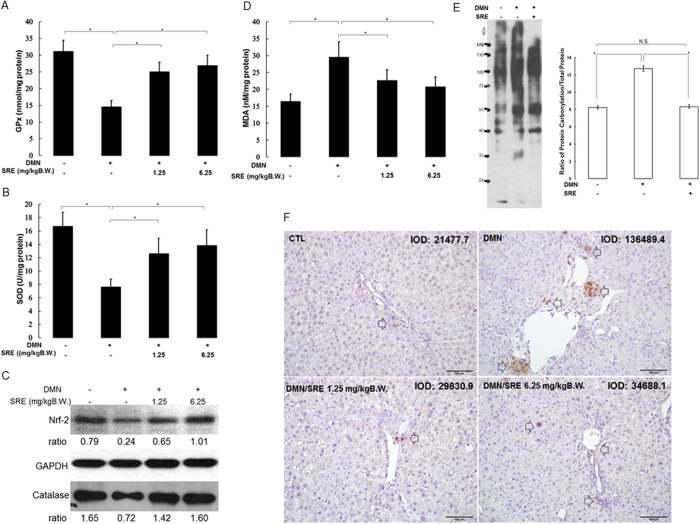
Effect of SRE on the GPx (**A**) and SOD (**B**) levels in control sample and DMN-treated liver tissues. (**C**) The levels of Nrf-2 and catalase under various treatments were performed by Western blot analysis. The relative expression to GAPDH is shown at the bottom. (**D**) Effect of SRE on the MDA production. (**E**) Expression of protein carbonylation. Significantly increased levels of carbonylated proteins were observed in the DMN group compared to the control, while SRE could effectively reduce the levels of carbonylated proteins. The quantified ratios are shown by the bar chart. (**F**) 8-OHdG levels were measured by immunocytochemistry. 8-OHdG-positive cells (indicated by arrow) were increased in the DMN treatment group compared to the normal control group. SRE decreased 8-OHdG levels in hepatic cells under DMN-induced oxidative stress. The data are the means ± S.D. (n = 3), and * indicates a significant difference (*p* < 0.05). The intensity was calculated using the Image Pro-Plus 4.5 computer program. IOD: integrated optical density.

Since DMN application catalyzes an excessive amount of ROS, we assessed the content of oxidative stress markers, including glutathione peroxidase (GPx) and superoxide dismutase (SOD) in the liver. Decreased hepatic GPx and SOD levels were observed in the DMN-treated subjects compared with the control group, while treatments with different doses of SRE could promote the liver GPx and SOD content, thus protecting the liver against ROS damage (Fig. 4A,B). To further reveal the molecular mechanism associated with regulation of the antioxidant enzymes, Nrf-2, which is a critical transcription factor as a major contributor to the activation of antioxidant system, was evaluated. DMN exposure remarkably suppressed Nrf-2 expression with respect to the control, while SRE treatment upregulated the level of Nrf-2. In line with this finding, an obvious decrease in catalase expression under DMN administration was observed. The catalase level was significantly restored in response to SRE in a dose-dependent manner (Fig. 4C). Compared with the control group, samples treated with DMN showed a twofold increase in the amount of MDA; however, SRE treatments markedly reversed the effect of DMN on MDA formation (Fig. 4D). Protein carbonylation and 8-hydroxydeoxyguanosine (8-OHdG) are considered to be two important parameters of oxidative stress. Fig. 4E demonstrates protein carbonylation in the normal and experimental liver tissue. The extent of protein carbonylation was significantly induced due to DMN exposure, compared with the normal tissue. However, post-treatment SRE decreased the levels of protein carbonylation in the hepatic tissue. In addition, oxidative modified DNA in the form of 8-OHdG can be quantified to indicate the extent of DNA damage. As shown in Fig. 4F, increasing amounts of 8-OHdG signal were detected in DMN-treated samples with respect to the control, whereas high and low doses of SRE caused a considerable reduction in 8-OHdG levels. These results suggest that SRE could effectively abolish DMN-mediated ROS generation as predicted by network analysis.

## Discussion

Liver fibrosis is a common and characteristic pathological feature of chronic liver diseases. Current treatment of liver fibrosis is hindered due to the lack of safe and effective agents for liver fibrosis therapy. Traditional Chinese medicine has been widely used in treating chronic liver diseases, and some herbal medicine and extracts have been confirmed to have antifibrotic effects^24^. In the present study, we investigated the inhibitory effects of SRE against DMN-induced liver fibrosis *in vivo*.

DMN is a potent hepatotoxin, carcinogen and mutagen that can result in various liver pathological characteristics, including fibrogenesis, parenchymal cell destruction, ascites, and nodular regeneration^25^,^26^. Based on our findings, SRE could significantly alleviate liver damage and reduce the accumulation of α-SMA, thereby protecting the liver from fibrogenesis under DMN stimulation. SRE administration significantly ameliorated elevated serum aminotransferases and bilirubin levels in a dose-dependent manner. Moreover, SRE application reduced the levels of PCNA, an indicator of increased proliferation, suggesting that SRE could exert a protective function against chemical-induced liver fibrogenesis.

As far as we know, DMN treatment leads to liver damage via the generation of oxidative stress, which has been well recognized as a critical contributor in the development of hepatic fibrogenesis^27^,^28^. It has been shown that SRE could induce Nrf-2, which activates the Nrf2–ARE pathway to stimulate the expression of multiple antioxidant enzymes^29^. As a result, SRE can prevent oxidative damage by elevating the levels of SOD, GPx and catalase in the DMN-exposed liver. In DMN-induced toxicity, radicals induced by DMN further attack cellular macromolecules such as lipids, proteins and DNA^30^,^31^,^32^. MDA, a major product of lipid peroxidation, may cause the loss of integrity of cell membranes and damage hepatic tissue concentrations. It was markedly increased under DMN application, whereas SRE caused a significant decrease in the DMN-elevated MDA level, suggesting effective protection against DMN-induced lipid peroxidation in the liver. Abundant evidence indicates that oxidation modification to proteins and DNA would lead to severe injury to tissues. According to the results, the most striking feature was that the protein and DNA were highly oxidized in the DMN-treated samples compared to the SRE-administrated group. Oxidation of protein and DNA manifested by protein carbonylation and 8-OHdG modification was also ameliorated by SRE treatment, which restored hepatic injury and improved organ functions. Moreover, several flavone components, including baicalin, wogonoside, baicalein and wogonin, were identified in the extract of the herbal combinatorial formulae. These bioactive compounds could effectively scavenge ROS in the process of neutralization and subsequently protect the essential thiol groups from oxidation^17^,^33^. Our findings imply that SRE might attenuate DMN-mediated liver injury through improving the antioxidant capacity by induction of the antioxidative enzymes and removal of ROS. The possible roles of SRE in the preventive effect against oxidative stress-induced injuries were verified. Meanwhile, inhibition of cell survival mediated by the active compounds of SRE was also evaluated by MTT assays, as given in the Supplement Table. In particular, emodin, gallic acid and baicalein resulted in more than 50% suppression in HSC-T6 cell growth at 50 μg/mL. Therefore, we applied the combinatorial formula to reduce the cytotoxicity of respective compounds. In this study, SRE at 6.25 mg/kg could be effective in protection against hepatic fibrogenesis; the proper dose for human application should be about 40 mg/kg, which is calculated based on the body surface area (BSA) method^34^. Although the positive effects of SRE on DMN-induced liver fibrosis, however, there are several reports indicated that some Chinese herbal medicine might cause to liver injury while our study does not provide the long-term safety of applying this new formula^20^,^35^,^36^. It is very important to take precautions against drug-induced liver damage for chronic use. This critical pending issue should be addressed in further investigation.

Potential drug targets and global cellular mechanisms can be addressed through analyses of differential protein profiles revealed by a functional proteomic tool^37^. Our results identified that 9 protein spots displayed significant changes in volume. After network analysis, these proteins could be grouped into several categories according to their known functions: oxidative stress-responded proteins, cytokeratin proteins and ER stress-associated proteins. A description of proteins that may be critical to liver fibrogenesis as well as numerous physiologic pathways is given as follows.

The first top-rank network is mainly responsible for oxidative stress. Several redox state-associated proteins, such as protein disulfide isomerase (PDI) and peroxiredoxin-2 (PRDX2), were significantly upregulated after DMN application, compared to the control group, whereas SRE treatment at low and high dose resulted in a remarkable reduction in protein levels. PDI is a redox-sensitive chaperone that participates in the processing of oxidized proteins and in preventing protein misfolding^38^,^39^,^40^. Therefore, it is reasonable that oxidative stress caused by DMN would promote the PDI expression level, whereas the administration of SRE could eliminate oxidative stress, resulting in a significant reduction of the PDI amount, as indicated in our proteome results.

Similarly, PRDX2 is a major antioxidant enzyme that requires NADPH to recycle its oxidized form. Several studies have indicated that PRDX2 plays a pivotal role in various cellular functions, including the protection of proteins and lipids against oxidative injury, as well as the mediation of signaling pathways associated with apoptosis^41^,^42^. Moreover, downregulation of PRDX2 sensitizes some cancer cells to radiation and chemotherapeutic drugs. Accordingly, PRDX2 overproduction caused by DMN should reflect an oxidative assault that results in liver damage, while Prd2 was significantly decreased under SRE treatment, suggesting that SRE could effectively prevent ROS production, and decompose ROS induced by DMN.

Given that cytoskeletal rearrangement is crucial for the epithelial-mesenchymal transition (EMT) during liver fibrogenesis, we also focused on the cytoskeleton group of proteins^43^. The proteomics and Western blot analyses indicated that DMN induced levels of several cytoskeletal proteins such as K8 keratins and β-tubulin, which is considered to be involved in the development and adverse outcome of multiple liver diseases. Adult hepatocytes are particularly unique in that they express K8 only; an increase in the keratin level is widely used as a noninvasive marker for liver injury. The reason for the toxic effect of keratin overexpression is likely associated with the keratin-related molecular events, including keratin hyperphosphorylation and keratin filament reorganization^44^. In this regard, SRE exposure appeared to alleviate the dysregulation of the cytokeratins, which may attenuate DMN-mediated liver damage. In addition, β-tubulin dramatically increased after treatment with DMN, which partially appeared to result in the disruption of microtubule dynamics, finally leading to cell apoptosis^37^. Again, the expression of β-tubulin was restored to a normal level under SRE application, implying that SRE could effectively protect hepatic tissue from DMN-induced damage.

**Figure 5:**
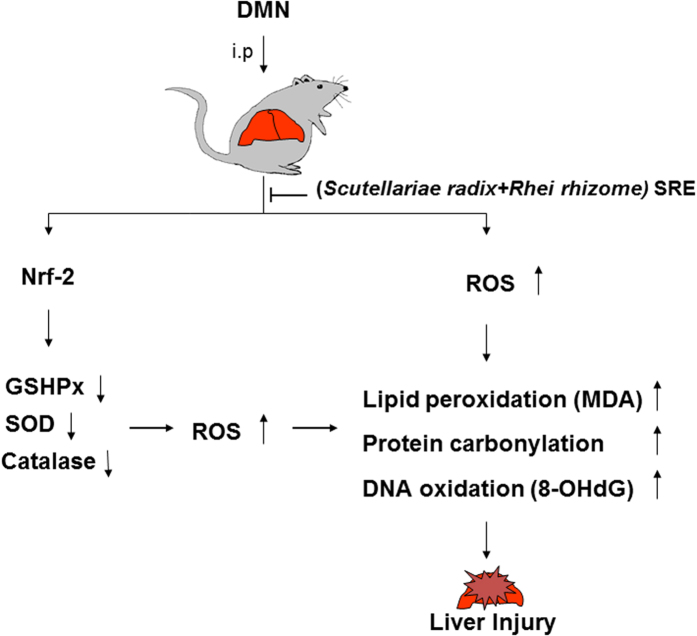
Schematic diagram of DMN-mediated liver injury through downregulation of antioxidant enzymes and inducing oxidative modification of biological molecules such as lipid, protein and DNA. SRE could effectively alleviate the hepatic damage via modulating the redox imbalance.

The current study elucidated a significant association between liver etiology and DMN. A large-scale comparison of protein profiles with or without SRE application in the presentation of DMN showed that SRE could exert liver-protective efficacy via its antioxidant characteristic, which consequently maintains the stability of biological molecules such as lipid, protein and DNA (Fig. 5).

## Materials And Methods

### Preparation of Scutellariae radix and Rhei rhizoma ethanolic extracts (SRE)

Scutellariae radix and Rhei rhizoma were purchased from a traditional Chinese medicine dispensary (Dan Yi pharmaceutical company, Taiwan). The voucher specimen (CGU-SRE) was deposited in the herbarium of Chang Gung University. The combination ratio was 1:2 for Scutellariae radix and Rhei rhizoma, according to the traditional Chinese medicine formula, and was extracted with ethanol. The ethanol-extracted solution was then concentrated to yield a yellow-brown syrup (extraction efficiency was approximately 25%) and was stored at −80 °C for use in all subsequent experiments. A high-performance liquid chromatographic method, coupled with ultraviolet (UV) rays, was performed for qualitative determination of compounds in the extract as follows: The samples were analyzed on a Hitachi L-2130 with wavelength 280nm, and the column used a Mightysil RP-18, 5 μm (250 × 4.6 mm) maintained at ambient room temperature. The gradient elution was performed using the mobile phase, with methanol as solvent A and water as solvent B. The gradient was as follows: 0 ~ 10 min, from 5% rising to 30% solvent A; 10 ~ 40 min, from 30% rising to 60% solvent A; 60 ~ 70 min, from 60% rising to 100% solvent A; 70 ~ 75 min, 100% solvent A. The flow rate was 0.7 mL/min. These compounds were identified by comparison of their retention time (Rt) with those of known standards from the chromatograms.

### Animal experiments

Specific pathogen-free 6-week-old male Wistar rats were purchased from a commercial animal breeder (BioLASCO, Taiwan). The rats were acclimated for 1 week and housed in an environmentally controlled room at 22 ± 2 ˚C, 55% ± 10% relative humidity, and in 12-hr light/dark cycles; they were fed commercial pellets and tap water *ad libitum*. The rats were randomly divided into four groups of three rats each (control: saline-treated, DMN-treated, DMN/SRE 1.25 mg/kg body weight (B.W.)-treated and 6.25 mg/kg B.W.-treated). The rats were intraperitoneally injected with DMN (Dr. Ehrenstorfer GmbH, Germany) at 10 mg/kg body weight for 3 consecutive days each week for 4 weeks, and the rats of the treatment groups were fed with SRE for three weeks through oral administration from the second week of DMN exposure, as described by Hsu, with some modification^9^. The Committee on Research Involving Animal Subjects of Chang Gung University, Taiwan, approved all experiments in compliance with the standards of Chang Gung University’s Committee for the Use and Care of Animals.

### Histology and Immunohistochemistry

A piece of liver tissue fixed with 10% neutral-buffered formalin was then embedded in paraffin and sliced into 5-μm sections that were stained with haematoxylin-eosin and Masson’s trichrome for a histological assessment. Immunohistochemstry with α–SMA (at a 1:100 dilution in PBS) and 8-OHdG (Santa Cruz, at a 1:100 dilution in PBS) were applied to the specimens, as previously described^45^. The histological changes were evaluated by using optical microscopy (Olympus BX51, Japan) in nonconsecutive, randomly chosen ×200 or ×400 histological fields. The digital photomicrographs were then processed with DP-72 (Olympus). Image-Pro® plus 4.5 (Media Cybernetics, USA) image analysis software was used to quantify image signals.

### Serum biochemical analysis

The levels of albumin, total bilirubin, AST, and ALT were determined in the serum using a colorimetric analyzer (Dri-Chem 3000, Fuji Photo Film, Japan).

### Measurement of GPx, SOD and MDA in liver tissues

Liver tissue was homogenized in lysis buffer, centrifuged at 10,000 g for 5 min at 4 ˚C, and the supernatants were collected. The activity of GPx and SOD and the production of MDA were measured according to our previously described protocol, respectively^46^ The protein concentrations were measured using the Bradford Protein Assay Kit (AMRESCO) to normalize the levels of GPx, SOD and MDA.

1. GPx activity was expressed as nanomoles reduced NADPH oxidized to NADP per minute per milligram of protein, with a molar extinction coefficient for NADPH at 340 nm of 6.22 × 10^6^ Lmol^−1^cm^−1^.
2. The activity of SOD was determined by inhibition of pyrogallol auto-oxidation. Changes in absorbance at 420 nm were recorded, and the activity was determined from a standard curve of percentage inhibition of pyrogallol auto-oxidation with known SOD activity.
3. The production of MDA was examined using the thiobarbituric acid reactive substances method (TBARS). Briefly, 0.2 g supernatant was mixed with thiobarbituric acid and then heated for 45 min at 100 ˚C. After cooling, n-butanol was added to the mixture, and the absorbance of the upper organic layer was measured at 535 nm with a spectrophotometer.

### Two-dimensional Polyacrylamide Gel Electrophoresis (2-D PAGE)

200 μg of lysates were solubilized in the rehydration buffer and then separated by an 18 cm IPG strip on the IPGphor III (GE Healthcare) in the first dimension. The running condition of the isoelectric focusing (IEF) was followed using our previous protocol^45^. Following IEF separation and equilibration, electrophoresis was carried out on 10% acrylamide gels (Bio-Rad) until the bromophenol blue dye front reached the bottom of the gel. The gels were identified using a silver stain, and scanned using an ImageScanner (GE Healthcare). Protein spots were quantified using the Prodigy SameSpots software (Nonlinear Dynamics, UK). The expression levels of protein spots dysregulated >1.5 fold with statistical significance (*p*-value <0.05) among control, DMN and DMN/SRE should be considered as proteins of interest. All experiments were repeated at least three times.

### In-gel Enzymatic Digestion and Mass Spectrometry

Silver-stained spots were excised and were destained by 1% potassium ferricyanide and 1.6% sodium thiosulfate. Then the proteins were reduced with 25 mM NH_4_HCO_3_ containing 10 mM dithiothreitol (DTT) at 56 °C and alkylated with 55 mM iodoacetamide at room temperature for 30 min. Then, the proteins were digested with 20 μg/mL trypsin at 37 °C overnight. After digestion, the tryptic peptides were acidified with 0.5% trichloroacetic acid (TCA) and loaded onto an MTP AnchorChip™ 600/384 TF. MS analysis was performed on an Ultraflex™ MALDI-TOF mass spectrometer (Bruker-Daltonik). Monoisotopic peptide masses were assigned and used for database searches with the MASCOT search engine (http://www.matrixscience.com). Search parameters were set as follows: maximum allowed peptide mass error of 50 ppm, and consideration of one incomplete cleavage per peptide^45^.

### Western blot analysis

Proteins were separated with 10% SDS-PAGE and transferred to a PVDF membrane. For protein carbonylation, SDS-PAGE was incubated in a solution of 2 N HCl with 10 mM 2,4-dinitrophenylhydrazie (DNPH) at 25 ˚C for 20 min. After the carbonyls derivatization step, gels were washed with 2 M Tris-base/30% glycerol for 20 min and then blotted onto a membrane^47^. Western blot analysis was performed using COMT, PCNA, β-tubulin, Nrf-2, GAPDH (Santa Cruz), K8 (Cell Signal), anti-DNP (dinitrophenyl) and α–SMA (Sigma) overnight at 4 °C. Blots were washed and incubated with HRP-labeled secondary antibody. Enhanced chemiluminescence was used for signal detection. The Western blot experiments were repeated in triplicate.

### Biological network analysis using MetaCore™

Applied MetaCore™ software (vers. 5.2 build 17389, GeneGo, USA) was used to reveal associated ontological classes and relevant pathways represented among the proteins identified by the 2-D and PMF^45^.

### Statistical analysis

All values were presented as the mean ± standard deviation (SD). Statistical analysis of the mean values was carried out with the ANOVA using SPSS software (SPSS Inc., USA). Differences were considered as significant at *p < 0.05.

## Additional Information

**How to cite this article**: Pan, T.-L. *et al.* Herbal formula, Scutellariae radix and Rhei rhizoma attenuate dimethylnitrosamine-induced liver fibrosis in a rat model. *Sci. Rep.* **5**, 11734; doi: 10.1038/srep11734 (2015).

## Supplementary Material

Supplementary Information

## Figures and Tables

**Table 1:** Effects of SRE on liver functions of rats treated with DMN.

Animal group	AST (U/L)	ALT (U/L)	Albumin (g/dL)	Total Bilirubin (mg/dL)	Hepatic collagen content, mg/g of dry-weight liver^a^
CTL	106 ± 8	35 ± 2	3.8 ± 0.1	0.8 ± 0.1	11.8 ± 2.2
DMN	1158 ± 25^**#**^	570 ± 10^**#**^	2.7 ± 0.2^**#**^	1.3 ± 0.1^#^	64.2 ± 4.8^**#**^
SRE(1.25 mg/kg B.W.)	114 ± 15*	44 ± 6*	3.7 ± 0.1*	0.6 ± 0.0*	10.8 ± 2.4*
SRE(6.25 mg/kg B.W.)	117 ± 22*	39 ± 12*	4.0 ± 0.1*	0.6 ± 0.0*	11.2 ± 3.2*
DMN + SRE(1.25 mg/kg B.W.)	189 ± 8*	68 ± 3*	3.7 ± 0.2*	1.1 ± 0.1	13.6 ± 2.1*
DMN + SRE(6.25 mg/kg B.W.)	150 ± 10*	60 ± 6*	3.9 ± 0.3*	0.9 ± 0.1*	14.2 ± 1.8*

AST: aspartate transaminase; ALT: alanine transaminase; T. Bilirubin: total bilirubin. CTL, control; DMN, Dimethylnitrosamine; Data are mean ± SD from three experiments. *p < 0.01 vs. DMN, and ^#^p < 0.01 vs. control, respectively.

^a^The level of collagen was determined with the Sircol collagen kit according to the manufacture’s instruction.

**Table 2:** List of identified protein spots.

Spot No.	Protein name	Accession number	Mw /pI	Score (coverage)	DMN	Fold change (verus CTL)		Function
						DMN/SRE(1.25 mg)	DMN/SRE(6.25 mg)	
1	Tubulin beta chain	Q6P9T8	50.22/4.79	121(44%)	5.4	1.2	1.4	Microtubules
2	PDI	P04785	57.38/4.87	164(64%)	9.2	1.2	1.1	Catalyzes the formation, breakage and rearrangement of disulfide bonds.
3	Cytokeratin-8	Q10758	52.68/5.49	180(53%)	4.2	1.4	1.1	Together with KRT19, helps to link the contractile apparatus to dystrophin
4	Sulfotransferase c1	P50237	35.87/6.09	136(64%)	−4.6	1.3	1.2	May be involved in the activation of carcinogenic hyroxylamines.
5	Catechol O-methyltransferase	P22734	24.96/5.11	90(53%)	4.2	1.3	1.2	Shortens the biological half-lives of certain neuroactive drugs, like L-DOPAl.
6	Peroxiredoxin-2	P35704	21.81/5.34	71(40%)	3.2	−1.2	1.7	Redox regulation of the cell
**7**	Glycine N-methyltransferase	P13255	32.93/7.10	88(57%)	−3.5	1.2	−2.2	Regulation of tissue concentration of AdoMet and of metabolism of methionine.
8	Glutathione S-transferase Mu 2	P08010	25.73/7.30	193(82%)	3.1	−1.1	1.2	Conjugation of reduced glutathione to a wide number of exogenous and endogenous hydrophobic electrophiles. The olfactory GST may be crucial for the acuity of the olfactory process.
9	Glutathione S-transferase Mu 1	P04905	25.84/8.27	66(35%)	−38	−0.9	1.3	Redox regulation of the cell
