# Supplementary material for: Herbal formula, Scutellariae radix and Rhei rhizoma attenuate dimethylnitrosamine-induced liver fibrosis in a rat model
Source: Sci Rep. 2015 Jul 2;5:11734. doi: 10.1038/srep11734 (PMC4488958; doi:10.1038/srep11734)
Supplement: Supplementary Information [file srep11734-s1.doc]

**Herbal formulae, *Scutellariae radix* and *Rhei rhizome* attenuate dimethylnitrosamine- induced liver fibrosis in a rat model**

Tai-Long Pan, Pei-Wen Wang, Chun-Hsun Huang, Yann-Lii Leu, Tung-Ho Wu, Yun-Ru Wu, Jyh-Sheng You

**Supplement Table.** **Effect of active constituents derived from SRE upon HSC-T6 cell viability**

| **Name** / **concentrations** | 1 μg/mL | 5 μg/mL | 10 μg/mL | 50 μg/mL |
| --- | --- | --- | --- | --- |
| Gallic acid | 106.8% | 111.6% | 102.6% | 37.9% |
| Baicalin | 103.6% | 103.9% | 95.3% | 80.9% |
| Wogonoside | 97.3% | 101.8% | 102.6% | 97.6% |
| Baicalein | 99.9% | 100.5% | 96.9% | 48.9% |
| Emodin | 105.7% | 107.2% | 91.2% | 23.8% |
| Wogonin | 104.1% | 107.7% | 106.2% | 92.6% |


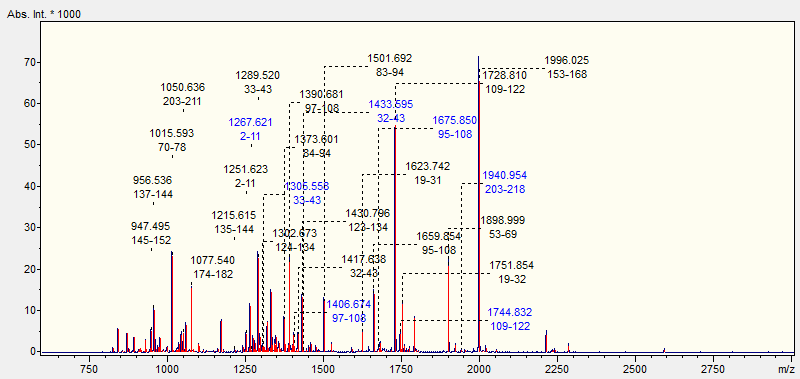

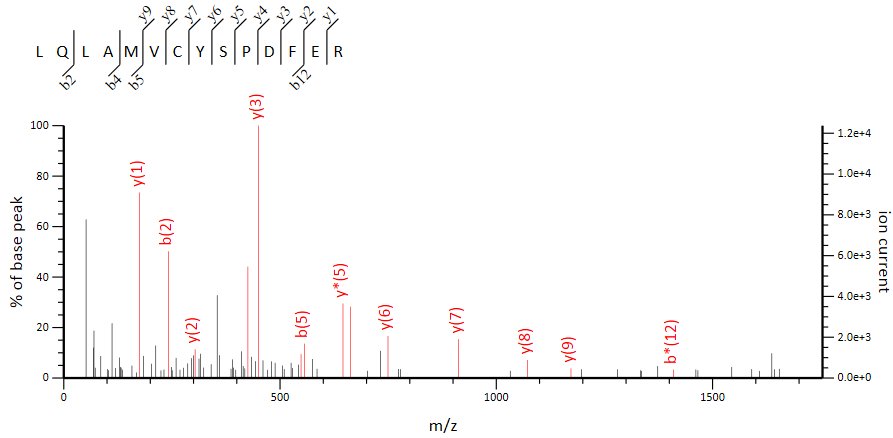


MS 1728.810


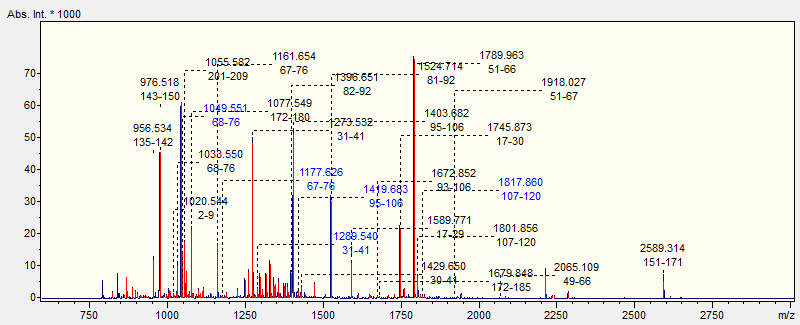


Spot 9 (GSTMu1)


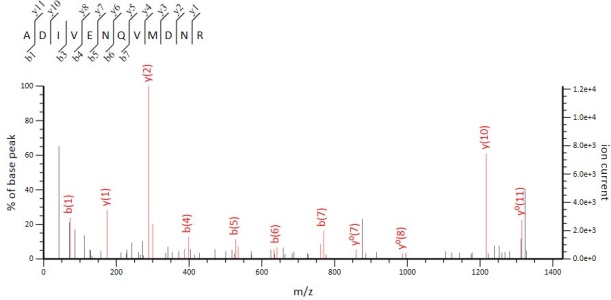


MS 1403.682

Supplement Figure 1S

Spot 8 (GSTMu2)

Supplement Figure 1S

A typical MALDI-TOF spectrum of trypsinized glutathione S-transferase Mu 2 and glutathione S-transferase Mu1. Their parent ions m/z 1728.810 and 1403.682 were selected for further analysis by an Ultraflex MS/MS operated in the LIFT mode using FlexControl software. A sequence was confirmed from the labeled b- and y-ions in the spectrum.
